# Three-dimensional organoid culture enhances functional maturation of human pluripotent stem cell–derived hepatocytes

**DOI:** 10.1007/s11033-026-12480-9

**Published:** 2026-07-29

**Authors:** Simona S. Ghanem, Tara Al-Barazenji, Rehab Badi, Essam M. Abdelalim

**Affiliations:** 1 Pluripotent Stem Cell Disease Modelling Lab, Translational Medicine Department, Research Branch, Sidra Medicine, P.O. Box 26999, Doha, Qatar; 2 College of Health and Life Sciences, Hamad Bin Khalifa University (HBKU), Qatar Foundation (QF), P.O. Box 34110, Doha, Qatar

**Keywords:** Induced pluripotent stem cells, Hepatic differentiation, Hepatocyte maturation, Transcriptomic profiling

## Abstract

**Background:**

Human pluripotent stem cells (hPSCs) offer a promising source of hepatocytes for disease modeling and drug screening. However, hepatocytes derived in conventional two-dimensional (2D) cultures often exhibit incomplete maturation, limiting their physiological relevance. The molecular mechanisms underlying the improved differentiation observed in three-dimensional (3D) culture systems remain poorly understood.

**Results:**

Induced PSCs (iPSCs) were differentiated into hepatic progenitors under 2D monolayer culture. At the hepatic progenitor stage, cells were either continued in 2D or re-aggregated into 3D organoids for hepatocyte maturation. Both conditions were cultured under identical experimental conditions throughout terminal differentiation for controlled comparison. At the end of differentiation, cultures were evaluated by immunostaining, gene and protein expression, functional assays, and RNA sequencing. Compared with 2D cultures, 3D hepatic organoids showed higher expression of mature hepatocyte markers (ALB, CPS1, AAT, and CYP3A4) and improved function, including increased albumin secretion, glycogen storage, and urea production. RNA sequencing identified 1,266 differentially expressed genes with upregulated genes enriched in hepatic metabolic pathways and downregulated genes associated with focal adhesion and extracellular (ECM)-receptor interaction.

**Conclusions:**

These findings demonstrate that 3D organoid culture enhances iPSC-derived hepatocyte maturation by activating liver-specific transcriptional programs, while suppressing progenitor-associated gene signatures, supporting its use for liver disease modelling and drug metabolism studies.

**Supplementary Information:**

The online version contains supplementary material available at 10.1007/s11033-026-12480-9.

## Background

The liver, the largest gland in the human body, plays a central role in regulating numerous physiological processes, including lipid and carbohydrate metabolism, glycolytic and urea metabolism, plasma protein synthesis, and the detoxification of a wide range of molecules [1]. Approximately 70–85% of the liver’s volume is composed of hepatocytes, which originate from the anterior portion of the definitive endoderm (DE). Hepatocytes are highly specialized cells that synthesize and secrete large amounts of proteins into the bloodstream, reflecting their critical roles in metabolic regulation and systemic homeostasis. These multifunctional properties highlight the importance of generating mature and functional hepatocytes in vitro to better model liver physiology and disease.

Hepatocytes derived from pluripotent stem cells (PSCs) represent a powerful and versatile model system for studying liver biology, disease mechanisms, and therapeutic applications [2, 3]. Human PSCs (hPSCs) possess a remarkable capacity for unlimited self-renewal and the potential to differentiate into any cell type of the human body, including liver cells [4, 5]. The advent of induced pluripotent stem cell (iPSC) technology has further expanded this potential by enabling the generation of patient-specific cell lines that capture individual genetic susceptibilities [6, 7]. Through established differentiation protocols, hPSCs have been directed toward hepatic lineages that exhibit essential hepatocyte characteristics. These advances have provided valuable in vitro platforms for modeling human development, drug metabolism, and liver-related diseases, as well as for screening hepatotoxic compounds [2, 3, 8]. However, many hPSC-derived hepatocytes exhibit incomplete maturation, with functional and transcriptional profiles that do not fully recapitulate the cellular and functional maturity of the human liver [9–11].

To address this limitation, we employed a well-established two-dimensional (2D) differentiation protocol with slight modifications [12, 13] and compared the outcomes of hepatocyte differentiation in conventional 2D monolayer with three-dimensional (3D) mature hepatocytes (3D-MH) under identical cytokine and media conditions. Our findings demonstrate that transitioning hepatic progenitors (HPs) from 2D to 3D culture markedly enhances hepatocyte maturation and functionality. Transcriptomic profiling further identified key signaling pathways associated with this improvement, highlighting molecular signatures associated with enhanced hepatic differentiation in 3D conditions.

## Materials and methods

### Culture and differentiation of iPSCs to hepatocytes

Established iPSC cell-line generated at our lab (Ctrl1-iPSCs) [14–18] were cultured using Stemflex (ThermoFisher Scientific, United States) media on Geltrex-coated plates (ThermoFisher Scientific, United States). Equal number of iPSCs were seeded and differentiated into mature hepatocyte-like cells (HLCs) using a previously published protocol [12, 13]. iPSCs were first differentiated into hepatic progenitor cells under 2D monolayer culture conditions. Upon completion of the hepatic progenitor stage, one group of cells was maintained under standard 2D maturation conditions while the remaining cells were dissociated into single cells using TrypLE Express (Thermofisher Scientific, United States), counted and seeded into Aggrewell 400, 24-well plates (Stem Cell Technologies, Canada) at a density of 2 × 10^6^ cells per well (~ 1,666 per microwell) to generate 3D-MH. After 48 h, 3D-MH were moved to ultra-low attachment plates on a shaker for further differentiation. Both groups were subsequently maintained using the same differentiation medium and culture conditions, allowing a controlled comparison of the effects of 2D versus 3D maturation. Differentiation reagents are listed in Supplementary Table 1.

### Immunofluorescence staining

For 2D cultured cells, immunostaining was performed on coverslip-grown cells fixed with 4% paraformaldehyde (PFA) in PBS for 25 min, following a previously described protocol [16]. Cells were washed with Tris-buffered saline (TBS) containing 0.5% Tween-20 (TBST) and then permeabilized in PBS with 0.5% Triton X-100 (PBST) for 2–3 h, then blocked overnight at 4 °C with 6% Bovine Serum Albumin (BSA) in 0.5% PBST. The percentage of double positive AFP/HNF4α cells were analyzed from three independent replicates using ImageJ. The percentage of AFP^+^/HNF4α^+^ cells was calculated relative to the total number of DAPI-positive nuclei. The results are shown as mean ± SD (*n* = 3). For 3D-MH, fixation with 4% PFA was extended to 30 min and permeabilization with 1% PBST was extended to two days at 4 °C and blocked overnight at 4 °C with 6% BSA in 1% PBST. Primary antibodies were applied overnight for 2D-MHs and two days for 3D-MH at 4 °C, followed by three TBST (0.5% Tween) washes. Secondary antibodies were added for 1 h for 2D-MHs and two hours for 3D-MH at room temperature, then washed again with TBST three times. Nuclei were stained with Hoechst 33,258 (1:10,000 in PBS). Coverslips were then mounted on slides using Diamond anti-fade mounting media (P36965, ThermoFisher Scientific, United States) and imaged on Zeiss LSM 780 Confocal Microscope at the Advanced Imaging Core (AIC) at Sidra Medicine. The details of the antibodies are listed in Supplementary Table 2.

### Western blotting

Protein extraction was performed by collecting cells from a single well of a 6-well plate and lysing with RIPA buffer (ThermoFisher Scientific, United States) supplemented with protease and phosphatase inhibitors (ThermoFisher Scientific, United States). Protein concentrations were determined using the Pierce BCA kit (ThermoFisher Scientific, United States). 20 µg of protein were loaded and separated on either 10% or 7% SDS-PAGE gels. The proteins were transferred to PVDF membranes (ThermoFisher Scientific, USA) via semi-dry transfer. Membranes were blocked in 10% skimmed milk in 0.5% TBST for 1 h at room temperature, then incubated overnight at 4 °C with primary antibodies. After washing with TBST, membranes were incubated with secondary antibodies for 1 h at room temperature, followed by additional washes with TBST. Detection was carried out using Supersignal West Pico or West Femto chemiluminescent substrates (34580 or 34096, ThermoFisher Scientific, United States). Protein expression was quantified by densitometry using ImageJ. Target protein intensity was normalized to the housekeeping gene, and values were expressed relative to the HP group, which was set to 1. Data are presented as mean ± SD from three independent biological replicates. Details of the antibodies used are provided in Supplementary Table 2.

### Total RNA extraction and RT-qPCR analyses

Cells from a 6-well plate were collected using 700 µL TRIzol Reagent (15596018, ThermoFisher Scientific, United States) and RNA was extracted with Direct-zol RNA Miniprep (R2052, Zymo Research, United States). RNA purity for all samples was assessed using spectrophotometric absorbance ratios, with A260/A280 values of approximately 2.0 and A260/A230 values ranging from 2.0 to 2.2. cDNA was synthesized from 1 µg total RNA following the High-Capacity cDNA Reverse Transcription Kit protocol (4374966, ThermoFisher Scientific, United States). Quantitative RT-PCR was performed using GoTaq qPCR SYBR Green Master Mix (A6110, Promega, United States). Statistical analyses for all RT-qPCR experiments were performed using ΔCt values. Relative gene expression is presented as fold change (2^-ΔΔCt). Analyses of mature hepatic markers by qPCR were performed on five independent biological replicates (*n* = 5), while qPCR experiments for RNA-seq validation was assessed on four independent biological replicates (*n* = 4). GAPDH served as the endogenous control; primer details are in Supplementary Table 3.

### Albumin ELISA

Cells at day 21 of hepatic differentiation from 2D and 3D-MH were cultured for 48 h without changing media, centrifuged at top speed and the supernatant was used to measure albumin content using the Human Albumin ELISA kit according to the manufacturer’s recommendations (ab108788, Abcam, United Kingdom). Albumin content was normalized to live cell count (*n* = 4).

### Glycogen assay

Glycogen content was quantified using Abcam’s Glycogen Assay (ab6520, Abcam, United Kingdom) according to the manufacturer’s instructions and normalized to live cell count (*n* = 4). Briefly, MHs at day 21 were harvested using TrypLE Express and resuspended in ice-cold water to disrupt cell membrane. Cells were counted, sonicated, denatured at 95 °C for 10 min, and centrifuged at 18,000 g for 10 min. Samples were then diluted at 1:5, and the assay was performed according to the manufacturer’s instructions.

## Urea assay

Urea was measured using Abcam’s Colorimetric Urea Assay kit (ab83362, Abcam, United Kingdom) according to the manufacturer’s instructions and normalized to live cell count (*n* = 4). Briefly, MHs at day 21 were harvested using TrypLE Express and resuspended in the supplied assay buffer. Cells were counted, sonicated, and centrifuged for 5 min at 4 °C at top speed. Samples were then diluted at 1:3 and the assay was performed according to instructions.

### RNA sequencing and bioinformatics

Next-generation RNA sequencing (RNA-seq) was carried out by the Genomics Core Facility at Sidra Medicine. RNA samples from four biological replicates per condition (2D-MH and 3D-MH) were used to generate sequencing libraries with the TruSeq Stranded mRNA Kit (Illumina), following the manufacturer’s protocol. Sequencing was performed on the Illumina NextSeq 500 platform, with paired-end reads of 42 bp × 42 bp per sample. Sequencing depth ranged from 26.8 to 36.6 million reads per sample (mean: 32.6 million).

Bioinformatics analysis was conducted using Partek Flow software (Partek Inc., St. Louis, MO, USA). Raw reads were aligned to the human genome (hg38) using STAR-2.7.3a. Uniquely mapped reads ranged from 71.22% to 78.55% across all samples (mean: 75.05%). Read assignment to annotated features was performed using feature counts, yielding 17.5–25.5 million assigned reads per sample (mean: 22.1 million; assignment rate: 64.6–72.0%). No samples were excluded from downstream analysis. All samples were processed in a single library preparation and sequencing run; therefore, no batch correction was applied.

Filtered raw counts were normalized by the addition of a pseudocount of 1.0, followed by Trimmed Mean of M-values (TMM) normalization and log₂ transformation. Differential expression analysis was performed using DESeq2; genes were considered differentially expressed at a False Discovery Rate (FDR)-adjusted p-value < 0.05 and log_2_ fold-change ≤ -1.0 or **≥** 1.0.

To assess replicate consistency and global transcriptomic variation between conditions, principal component analysis (PCA) was performed on the rlog-normalized expression matrix. PCA scores were plotted using the ImageGP platform [19] (https://www.bic.ac.cn/ImageGP), and color-coded by condition. To statistically assess whether the condition explained a significant proportion of transcriptomic variance, a permutational multivariate analysis of variance (PERMANOVA) and a corresponding homogeneity of multivariate dispersions test (betadisper) were performed directly within the ImageGP pipeline, utilizing its underlying R vegan package backend. This ensured that any significant PERMANOVA result reflected true structural differences between group centroids rather than artifacts of unequal within-group variance.

Heatmaps were generated using the Morpheus web application (Broad Institute; https://software.broadinstitute.org/morpheus*).* For the global expression heatmap, the top 1,000 most variable genes were selected based on calculated feature variance. Expression values were transformed to row Z-scores to highlight relative expression changes across groups. Unsupervised hierarchical clustering was applied simultaneously to rows (genes) using Euclidean distance and complete-linkage method. The color scale was fixed between Z-scores of − 2 and + 2.

Pathway enrichment analysis was performed using Gene Set Enrichment Analysis (GSEA) with KEGG pathway gene sets through the WEB-based GEne SeT AnaLysis Toolkit (WebGestalt; https://www.webgestalt.org) [20]. The complete ranked gene list, ordered by log₂ fold-change (Supplementary Table 4), was used as input against KEGG pathway gene sets. Pathways with FDR q-value < 0.05 were considered statistically significant.

### Transcriptomic comparison of iPSC-derived hepatocytes and human liver tissue

To benchmark iPSC-derived hepatocytes against primary human liver tissue, our RNA-seq data from 2D-MH (*n* = 4) and 3D-MH (*n* = 4) were integrated with publicly available human liver RNA-seq data (*n* = 6) from the Genotype-Tissue Expression (GTEx) portal [21] . The GTEx raw counts were processed using the same Partek Flow pipeline applied for our RNA-seq data, comprising sequential steps of quantification, feature filtering, batch effect removal, and TMM normalization, to ensure comparability across datasets. Batch effect removal was performed using the ComBat algorithm within Partek Flow to account for technical variation introduced by integrating data from different sources. Normalized gene expression counts were then exported and scaled as Z-scores for visualization. Hierarchical clustering was performed on normalized counts across all three groups. Furthermore, a panel of 82 well-known mature hepatocyte marker genes was examined across the three groups to assess the hepatocyte maturity of 3D-MH and 2D-MH relative to primary human liver tissue.

### Statistical analysis

Biological replicates (four for RNA sequencing and qPCR validation experiments, five for hepatic mature RT-qPCR analyses, and four for functional assays) were included. Biological replicates represent independent differentiation experiments performed separately under the same experimental conditions. Statistical analysis was conducted using an unpaired two-tailed Student’s *t*-test with Prism version 8 (GraphPad Software, USA, www.graphpad.com*).* Results are presented as mean ± SD.

## Results

### Generation of mature hepatocyte-like cells (HLCs) from iPSCs in 2D and 3D culture systems

**Fig. 1:**
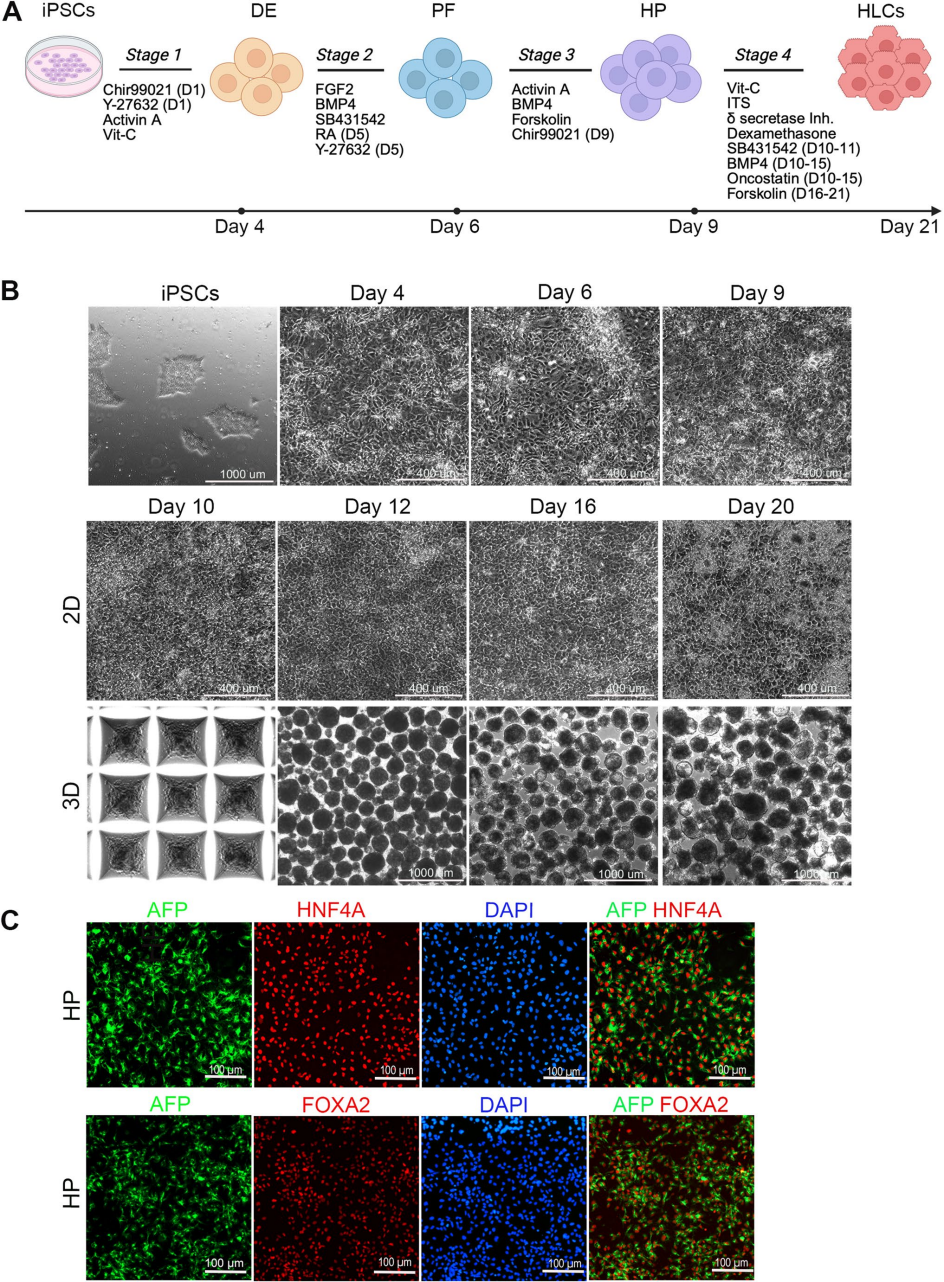
Differentiation of iPSCs into hepatocytes under 2D and 3D culture conditions. (**A**) Diagram illustrating the stepwise differentiation of iPSCs into hepatocyte-like cells (HLCs). (**B**) Morphological features at different stages of hepatocyte differentiation using two culture approaches (2D and 3D). At day 10 of differentiation, one group of cells were seeded for 48 h into AggreWell 400 plates to promote self-aggregation and three dimensional mature hepatocytes (3D-MH) while the other group continued as 2D culture. (**C**) Immunostaining showing the expression of hepatic progenitor (HP) markers, AFP, FOXA2, and HNF4A, at the end of HP stage. (Day 9). DE, Definitive Endoderm; PF, Posterior Foregut; HP, Hepatic Progenitor; HLCs: Hepatocyte-like cells

To investigate the impact of transitioning from 2D to 3D culture conditions after the hepatic progenitor (HP; hepatoblast) stage on the maturation and function of iPSC-derived HLCs, we employed a well-established hepatic differentiation protocol [12] with minor modifications [13] (Fig. 1A). Initially, iPSCs were differentiated into HPs under 2D culture conditions, achieving high differentiation efficiency (Fig. 1B, C). At day 10 of differentiation, the generated HPs were characterized using immunostaining, which confirmed robust expression of key HP markers such as alpha-fetoprotein (AFP), hepatocyte nuclear factor 4 alpha; HNF4A (HNF4A), and forkhead box A2 (FOXA2) [1, 13, 22] (Fig. 1C). Quantitative analysis of AFP^+^/HNF4A^+^ cells showed a value of 91.8 ± 2.1 (mean ± SD, *n* = 3) based on three independent measurements, indicating a highly enriched hepatic lineage population.

To assess the impact of 3D culture on hepatocyte maturation, HPs generated in 2D were further differentiated under either continued 2D conditions or 3D conditions to form MHs (Fig. 1B). At day 10 of differentiation, cells were seeded for 48 h into AggreWell 400 plates to promote organoid formation and 3D-MH. Over the following days, cells assembled into compact spheroidal structures typical of hepatic organoids by day 12 (Fig. 1B). By the end of differentiation (days 20 and 21), these organoids exhibited a stable architecture and maintained structural integrity, confirming successful establishment of 3D-MH suitable for functional and molecular analyses (Fig. 1B). On the other hand, 2D-MH displayed typical hepatocyte-like polygonal morphology with well-defined cell borders (Fig. 1B), highlighting distinct differences in cell organization between the two culture systems. Additionally, quantitative analysis of organoid diameter from fifteen 3D-MH per group demonstrated no significant differences across days 12, 16 and 20, indicating that organoid size remained stable throughout the 3D culture period (Supplementary Fig. 1).

### Enhanced maturation and functional properties of iPSC-derived hepatocytes in 3D organoids compared to 2D culture

**Fig. 2:**
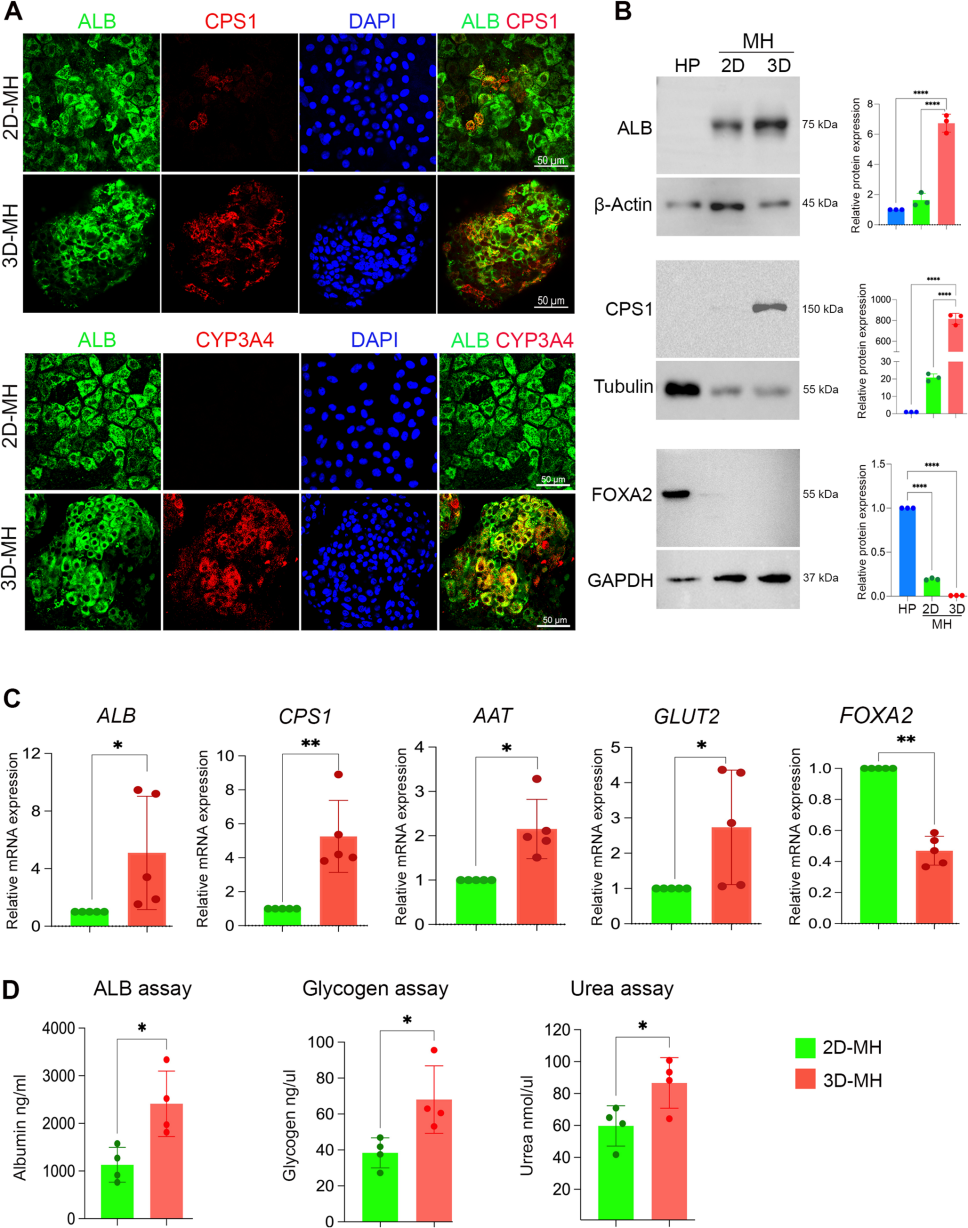
Transition from 2D to 3D culture enhances hepatocyte maturation and functional capacity. (**A**) Immunofluorescence analysis showing expression of mature hepatocyte markers, including ALB, CPS1, and CYP3A4. Note the higher expression of CPS1 and CYP3A4 in 3D-MH compared to 2D-MH. Hoechst was used for nuclear staining. (**B**) Western blot analysis demonstrating a substantial increase in ALB and CPS1 expression in 3D-MH compared to 2D-MH. FOXA2, a hepatic progenitor (HP) marker, is completely absent in 3D-MH, whereas low levels are detected in 2D-MH (*n* = 3). (**C**) RT-qPCR analysis showing upregulation of mature hepatocyte markers (*ALB*,* CPS1*,* AAT*,* GLUT2*) and downregulation of the HP marker *FOXA2* (*n* = 5). (**D**) Hepatic functional assays show increased ALB secretion, glycogen accumulation, and urea production in 3D-MH (*n* = 4). Data are presented as mean ± SD and normalized to live cell count. *p < 0.05, **p < 0.01, ***p < 0.001

To compare hepatic maturation at the terminal differentiation stage (day 21), iPSC-derived hepatocytes were analyzed in both 2D-MH and 3D-MH (Fig. 2). Immunostaining for key hepatic markers, including ALB, CPS1, and CYP3A4, revealed markedly stronger CPS1 and CYP3A4 signals in 3D-MH compared to 2D-MH (Fig. 2A), suggesting that 3D culture may contribute to improved maturation and functional capacity of iPSC-derived HLCs. Consistent with the immunostaining results, ALB and CPS1 protein levels, as assessed by western blotting (Fig. 2B), were higher in 3D-MH compared to 2D-MH, suggesting the potential of 3D culture conditions to promote hepatic maturation. In contrast, FOXA2, which is highly expressed in HPs but absent in MH [13, 23], was undetectable in 3D-MH and showed low expression in 2D-MH, potentially reflecting progression through hepatic differentiation toward a more mature hepatocyte phenotype in 3D culture (Fig. 2B). In parallel, quantitative gene expression analysis revealed significant upregulation of key mature hepatic genes, including *ALB*,* CPS1*,* AAT*, and *GLUT2*, in 3D-MH compared with 2D-MH (Fig. 2C), further supporting enhanced hepatic differentiation under 3D conditions. In contrast, expression of the HP marker FOXA2 was markedly reduced in 3D-MH organoids (Fig. 2C), in line with acquisition of a more differentiated hepatocyte phenotype toward a more mature hepatocyte-like phenotype. Taken together, these findings are consistent with the possibility that 3D culture may facilitate aspects of iPSC-derived hepatocyte maturation compared with conventional 2D differentiation.

To assess hepatic functionality at the mature stage (day 21), we assessed albumin secretion, urea synthesis, and glycogen storage. ELISA analysis showed that 3D-MH secreted significantly higher levels of albumin compared with 2D-MH, with a 2.2-fold increase (Fig. 2D), consistent with the elevated protein and gene expression of ALB observed in 3D organoids. Glycogen assays revealed a 1.5-fold higher glycogen content in 3D-MH compared to 2D-MH, indicating enhanced metabolic function and glucose storage. Furthermore, urea production was approximately 1.4-fold higher in 3D-MH (Fig. 2D), reflecting increased activity of the urea cycle. These functional improvements correlated with upregulated expression of key hepatic enzymes, including CPS1, as confirmed by immunostaining, western blot, and qRT-PCR (Fig. 2A–C), supporting a more mature HLC phenotype in 3D culture.

### Genome-wide transcriptomic analysis reveals pathways associated with enhanced maturation in 3D-cultured iPSC-derived hepatocytes

**Fig. 3:**
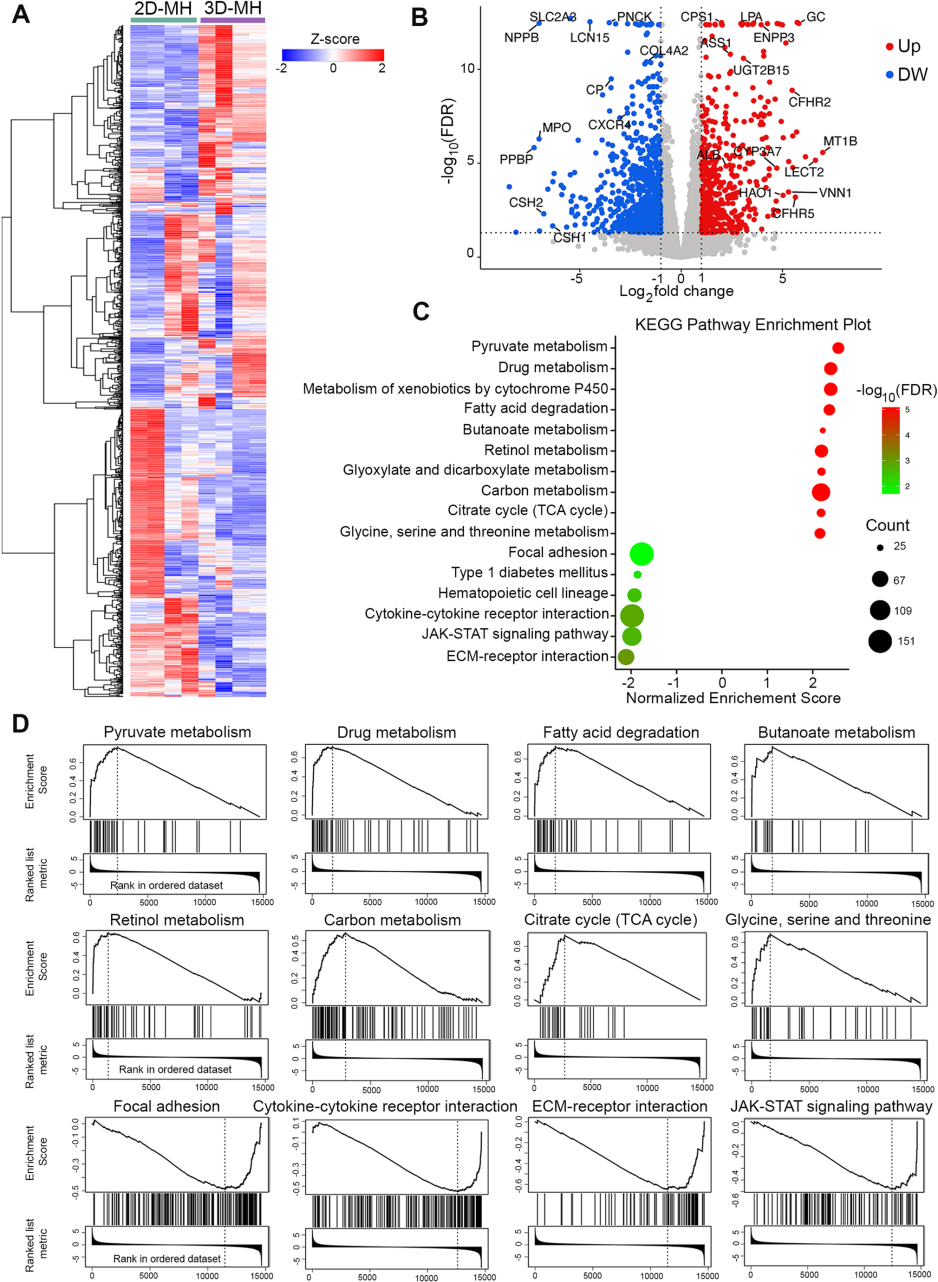
RNA-seq analysis of hepatocyte-like cells (HLCs) derived from 3D and 2D cultures. (**A**) Hierarchical clustering heatmap of differentially expressed genes (DEGs) between 2D-MH and 3D-MH culture conditions, shown as Z-scores (red: upregulated; blue: downregulated). (B) Volcano plot illustrating differentially expressed genes (DEGs) between 3D-MH and 2D-MH in iPSC-derived hepatocytes (FDR < 0.05) (*n* = 4). Red dots indicate upregulated genes (UP), while blue dots represent downregulated genes (DW) in 3D-MH compared to 2D-MH. **(C)** Kyoto Encyclopedia of Genes and Genomes (KEGG) pathway enrichment dot plot. Dot color represents − log₁₀(FDR) and dot size represents gene count. Positive and negative normalized enrichment scores (NES) indicate enrichment in 3D-MH and 2D-MH, respectively. **(D)** GSEA plots for key enriched KEGG pathways, including metabolic pathways enriched in 3D-MH and signaling/adhesion pathways enriched in 2D-MH

To better understand how the transition from 2D to 3D culture conditions impacts hepatocyte development and function, we performed bulk RNA-seq at the mature stage, and the differential gene expression analysis using DESeq2 was performed on samples from 3D-MH and compared to those from 2D-MH. PCA revealed clear transcriptomic separation between 2D-MH and 3D-MH conditions, with PCA1 and PCA2 collectively capturing 63.6% of the total variance (Supplementary Fig. 2). To examine global transcriptomic differences between 2D-MH and 3D-MH, unsupervised hierarchical clustering was carried out. The resulting heatmap showed a distinct separation between the two conditions (Fig. 3A), indicating that 3D culture exerts a substantial influence on the hepatocyte transcriptomic profile compared with 2D condition. A total of 1,266 differentially expressed genes (DEGs) were identified using a cutoff of log2 fold change (FC) ≤ -1.0 or ≥ 1.0 and FDR < 0.05. Among these, 477 DEGs were upregulated and 789 DEGs were downregulated (Fig. 3B). The top upregulated and downregulated genes are listed in Table 1.

**Table 1:** Top 50 upregulated and 50 downregulated differentially expressed genes (DEGs) in 3D-MH compared to 2D-MH (log₂FC ≥ 1.0 or ≤ − 1.0, FDR < 0.05)

Upregulated			Downregulated		
Gene ID	Log₂FC	FDR	Gene ID	Log₂FC	FDR
*GC*	5.81	5.30E-22	*NPPB*	-7.012	1.70E-24
*ADH1A*	5.73	5.60E-32	*SLC2A3*	-5.464	7.75E-146
*CFHR2*	5.48	1.31E-09	*LCN15*	-4.499	1.32E-40
*PAX4*	5.17	3.95E-12	*MS4A18*	-3.879	2.28E-09
*SLCO1B1*	5.00	3.54E-13	*PNCK*	-3.554	3.43E-29
*AKR1C1*	4.85	1.53E-18	*ERVH48-1*	-3.460	9.53E-10
*CPB2*	4.74	2.79E-21	*CP*	-3.451	3.21E-10
*ADH6*	4.64	3.86E-09	*SLC2A5*	-3.229	1.23E-18
*HAMP*	4.37	4.79E-10	*EGLN3*	-2.642	1.22E-11
*ARG1*	4.29	5.22E-09	*DUOX2*	-2.641	2.39E-19
*UGT2B17*	4.26	8.92E-21	*PRTG*	-2.430	8.61E-10
*BHMT*	4.08	1.97E-11	*SPAG4*	-2.394	2.65E-09
*GUCA2B*	4.07	1.11E-11	*GABRA2*	-2.254	1.52E-20
*ENPP3*	3.95	2.67E-14	*FGF23*	-2.125	1.01E-13
*MT1E*	3.92	3.07E-09	*HAPLN3*	-2.110	1.34E-14
*GCG*	3.58	2.17E-25	*HLA-DRB5*	-2.108	4.73E-10
*UGT2B4*	3.58	4.73E-09	*VLDLR*	-2.051	7.62E-13
*CYP4A11*	3.54	9.63E-10	*ARHGAP29*	-1.948	5.95E-10
*CD4*	3.53	4.59E-16	*HLA-DRB1*	-1.936	2.79E-21
*HAL*	3.30	2.13E-15	*PDK1*	-1.925	1.12E-22
*REP15*	3.27	2.18E-16	*PFKP*	-1.786	5.17E-11
*UGT2B15*	3.08	2.60E-11	*PTN*	-1.768	4.42E-10
*PON1*	3.04	2.12E-20	*FRMD4A*	-1.716	1.43E-12
*AGMO*	3.00	5.72E-13	*COL4A2*	-1.713	3.99E-11
*LPA*	2.95	1.49E-20	*SLFN13*	-1.705	1.01E-13
*NECAB1*	2.69	2.09E-09	*HILPDA*	-1.668	1.17E-12
*AADAC*	2.46	1.30E-10	*ZNF550*	-1.629	3.01E-10
*ASS1*	2.43	1.57E-11	*SLC6A8*	-1.581	8.61E-10
*OTC*	2.40	1.66E-10	*ABCA2*	-1.551	3.13E-17
*ALDH1L1*	2.30	9.53E-10	*TRIM6*	-1.547	2.62E-09
*DPYD*	2.16	6.73E-12	*PRSS12*	-1.537	1.30E-13
*TFF3*	2.03	8.21E-13	*PROM1*	-1.536	3.99E-09
*LRRC31*	2.00	9.41E-16	*COL4A6*	-1.505	4.37E-11
*CPS1*	1.99	1.93E-30	*SLC7A1*	-1.505	1.70E-14
*PLG*	1.85	2.39E-10	*ANKS1A*	-1.453	9.48E-20
*ACADSB*	1.69	2.16E-13	*FZD7*	-1.417	8.37E-10
*SERPINA5*	1.68	1.16E-09	*SLC4A4*	-1.395	1.82E-09
*ABAT*	1.67	1.63E-16	*BARD1*	-1.385	2.26E-10
*MSRB1*	1.54	4.93E-09	*DBF4B*	-1.384	2.50E-10
*HYAL1*	1.53	1.72E-12	*SLC25A29*	-1.352	8.37E-10
*DHRS7*	1.51	6.02E-09	*PTPN14*	-1.250	1.90E-11
*PBLD*	1.45	2.49E-10	*ZNF714*	-1.185	8.48E-10
*HADH*	1.40	2.67E-14	*MDC1*	-1.163	1.74E-10
*ADI1*	1.35	2.13E-10	*RBM3*	-1.147	4.81E-09
*FAHD1*	1.35	3.29E-09	*OTUD3*	-1.143	2.60E-09
*HSD17B11*	1.25	2.38E-14	*SEZ6L2*	-1.108	2.00E-14
*MT-ND3*	1.23	2.28E-11	*UNC13C*	-1.074	3.21E-10
*NDRG2*	1.22	5.83E-15	*GTPBP3*	-1.033	3.50E-10
*TMEM251*	1.18	2.93E-12	*COL4A5*	-1.025	1.86E-11
*SDHB*	1.05	8.99E-09	*CPE*	-1.000	5.51E-11

For pathway analysis, we performed GSEA on RNA sequencing data, with pathway annotations derived from the KEGG database. The results showed distinct and biologically coherent patterns of pathway enrichment and suppression in 3D-MH relative to 2D-MH. Multiple metabolic pathways were significantly upregulated in 3D-MH, as indicated by positive Normalized Enrichment Scores (NES) and high statistical significance (-Log_10_ FDR) (Fig. 3C, D). These included pyruvate metabolism, drug metabolism, metabolism of xenobiotics by cytochrome P450, fatty acid degradation, butanoate metabolism, retinol metabolism, glyoxylate and dicarboxylate metabolism, carbon metabolism, citrate cycle (TCA cycle), and glycine, serine and threonine metabolism. The enrichment of these pathways collectively suggests enhanced mitochondrial activity, oxidative metabolism, and hepatic detoxification capacity in 3D-MH, which are hallmarks of functionally HLCs. In contrast, other pathways were significantly downregulated in 3D-MH. These included focal adhesion, type 1 diabetes, hematopoietic cell lineage, cytokine–cytokine receptor interaction, JAK–STAT signaling pathway, and ECM–receptor interaction (Fig. 3C, D). These results suggest that 3D-MH drives a transcriptional shift toward a more metabolically active and functionally mature hepatocyte phenotype.

**Fig. 4:**
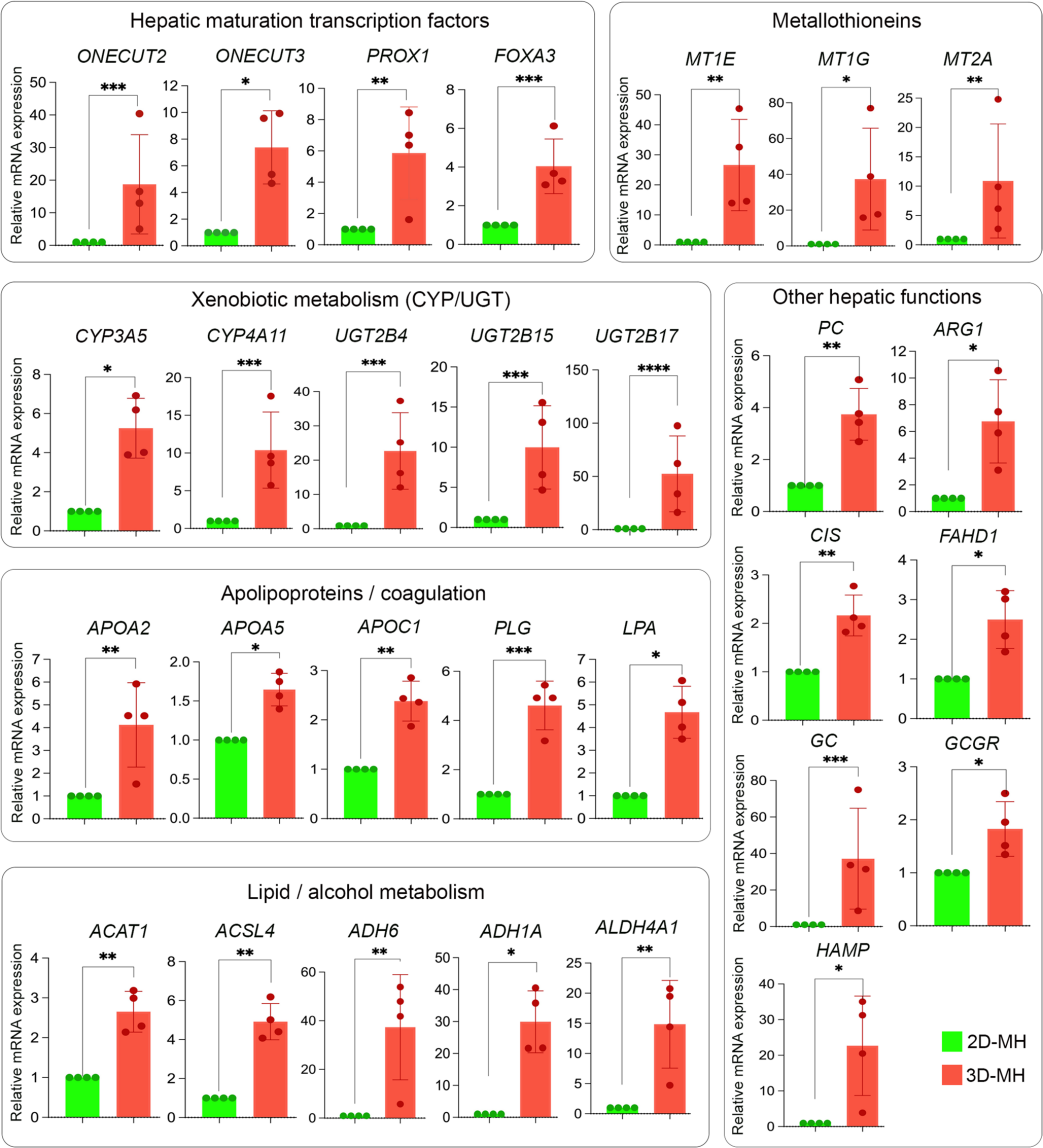
Validation of RNA-seq results confirms upregulation of key hepatic genes in 3D- cultured hepatocyte-like cells (HLCs). RT-qPCR analysis demonstrating increased expression of selected upregulated genes associated with hepatic development maturation in 3D-MH compared with 2D-MH (*n* = 4). The genes represent key functional categories, including hepatic maturation transcription factors, metallothioneins, xenobiotic metabolism, apolipoproteins and coagulation, lipid and alcohol metabolism, and other hepatic-specific functions. Data are presented as mean ± SD; *p < 0.05, **p < 0.01, ***p < 0.001

**Fig. 5:**
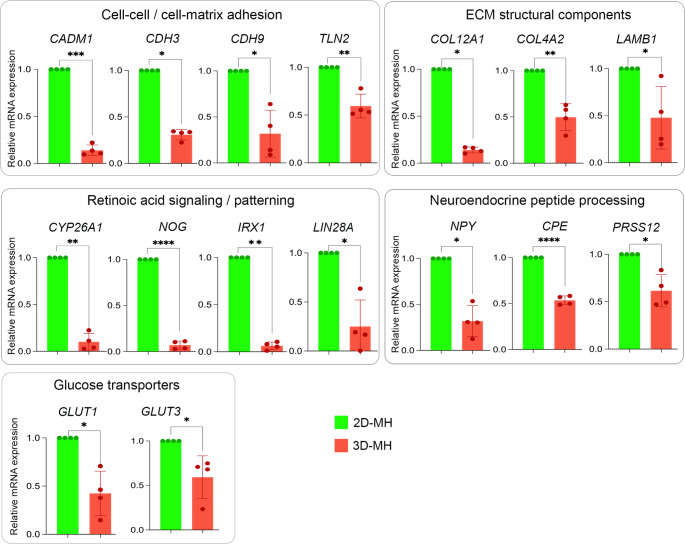
Validation of RNA-seq results confirms gene downregulation in 3D-cultured hepatocyte-like cells (HLCs). RT-qPCR analysis showing expression of selected downregulated genes in 3D-MH compared to 2D-MH (*n* = 4). The genes are associated with cell-cell and cell matrix adhesion, ECM structural componenets, retinoic acid signaling and developmental patterning, glucose transporters, and neuroendocrine peptide processing. Data are presented as mean ± SD; *p < 0.05, **p < 0.01, ***p < 0.001

Furthermore, RT-qPCR analysis of selected upregulated DEGs confirmed significant increase in the expression of many genes in 3D-MH compared to 2D-MH, including hepatic maturation transcription factors (*ONECUT2*,* ONECUT3*,* PROX1*,* FOXA3)*, metallothioneins *(MT1E*,* MT1G*,* MT2A)*, xenobiotic metabolism (*CYP3A5*,* CYP4A11*,* UGT2B4*,* UGT2B15*,* UGT2B17)*, apolipoproteins and coagulation *(APOA2*,* APOC1*,* APOA5*,* PLG*,* LPA)*, lipid and alcohol metabolism *(ACAT1*,* ACSL4*,* ADH6*,* ADH1A*,* ALDH4A1)*, and other hepatic-specific functions *(PC*,* ARG1*,* CIS*,* FAHD1*,* GC*,* GCGR*,* HAMP) *(Fig. 4). On the other hand, validation of several downregulated DEGs revealed significant reduction in the expression of genes associated with cell-cell and cell matrix adhesion *(CADM1*,* CDH3*,* CDH9*,* TLN2)*, ECM structural componenets *(COL12A1*,* COL4A2*,* LAMB1)*, retinoic acid signaling and developmental patterning *(CYP26A1*,* NOG*,* IRX1*,* LIN28A)*, glucose transporters (*GLUT1*,* GLUT3)*, and neuroendocrine peptide processing *(CPE*,* NPY*,* PRSS12)* (Fig. 5).

### 3D culture conditions enhance liver-specific gene expression relative to 2D cultures

**Fig. 6:**
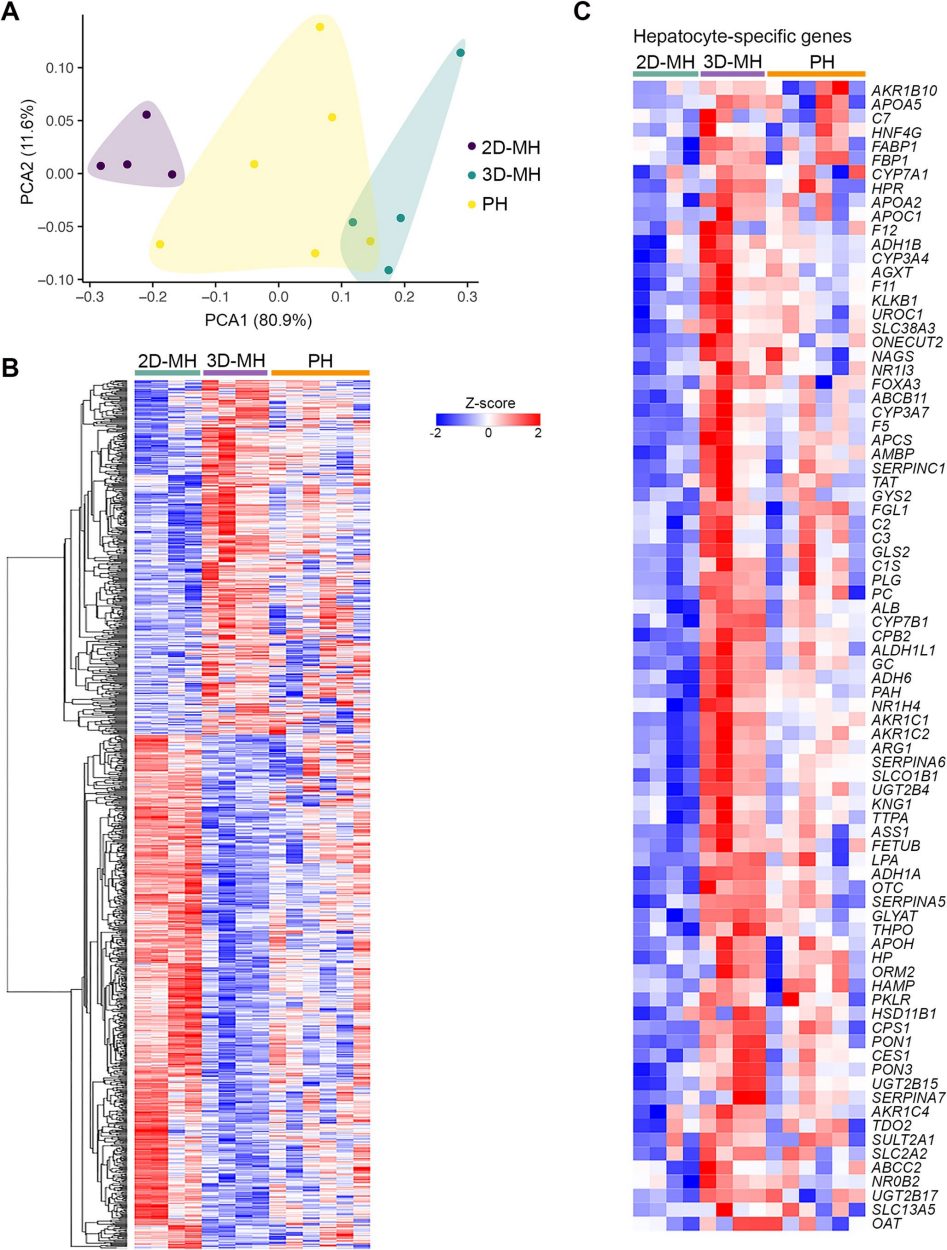
Transcriptomic comparison of iPSC-derived hepatocytes and primary human liver tissue. **(A)** Principal component analysis (PCA) of rlog-normalized gene expression data from 2D-MH (*n* = 4), 3D-MH (*n* = 4), and human liver tissue (PH) (*n* = 6), color-coded by condition. Each dot represents one biological replicate. (**B**) Hierarchical clustering heatmap of normalized gene expression counts across 2D-MH, 3D-MH, and human liver tissue (PH). Expression values are shown as Z-scores (scale: −2 to + 2; red: high expression, blue: low expression). **(C)** Hierarchical clustering heatmap of 82 well-known mature hepatocyte marker genes. The heatmap illustrates that the expression of these markers in 3D-MH closely resembles that of PH

To assess the maturity of iPSC-derived hepatocytes, normalized gene expression profiles of 2D-MH and 3D-MH were compared against human liver tissue from the GTEx database (Fig. 6A). Hierarchical clustering of normalized counts revealed distinct expression patterns across the three groups, with 3D-MH displaying an overall transcriptomic profile more similar to GTEx liver samples than to 2D-MH (Fig. 6A, B). To further evaluate hepatocyte maturity, we examined the expression of 82 well-known mature hepatocyte marker genes across all three groups. Interestingly, all 82 genes were identified as DEGs upregulated in 3D-MH compared to 2D-MH, and their expression in 3D-MH was broadly comparable to that observed in primary hepatocytes (Fig. 6C; Table 1). The 3D-MH exhibited the highest expression across the majority of mature hepatocyte markers, including key genes involved in drug metabolism (*CYP3A4* and *CYP7A1*), urea cycle function (*ARG1*,* ASS1*,* CPS1*,* OTC*), lipid and lipoprotein metabolism (*ALB*,* APOA2*,* APOA5*,* APOC1*, and *APOH*), bile acid synthesis and transport (*ABCB11*,* NR1H4*, and *CYP7B1*), and coagulation factor production (*F5*,* F11*,* F12*, and *SERPINC1*) (Fig. 6C). Interestingly, some 3D-MH samples showed expression levels that matched or exceeded those observed in primary human liver tissue, suggesting a high degree of hepatocyte maturation in these organoid cultures. In contrast, the 2D-MH showed markedly lower expression across nearly all mature hepatocyte markers, indicating that 2D culture conditions are associated with a less mature hepatocyte transcriptomic profile compared to 3D-MH (Fig. 6B, C). Collectively, these findings demonstrate that 3D culture conditions drive a more mature hepatocyte transcriptomic program in iPSC-derived HLCs, more closely recapitulating the gene expression profile of primary human liver tissue.

## Discussion

The generation of functional hepatocytes from hPSCs has emerged as a powerful platform for disease modeling, drug discovery, and potential cell-based therapies. However, a major limitation of conventional 2D culture systems is the incomplete maturation of iPSC-derived HLCs, which often fail to fully recapitulate the functional and molecular characteristics of adult liver tissue. While multiple studies have generated iPSC-derived HLCs cultured in 3D system, demonstrating that 3D systems promote enhanced hepatic maturation and functionality [24–28], most prior comparisons have focused predominantly on phenotypic and biochemical functional assays with limited integration of comprehensive transcriptomic profiling to elucidate the molecular mechanisms underlying 3D-induced maturation. The present study addresses this critical gap by uniquely integrating multiple complementary approaches, mature hepatic marker expression, functional assays, and comprehensive transcriptomic profiling, to provide a holistic and mechanistic understanding of how 3D architecture drives hepatocyte maturation. This integrative strategy not only confirms enhanced functionality in 3D organoid culture but also reveals the global gene expression programs and specific metabolic pathways activated during this transition, offering unprecedented molecular insights into the biological processes that govern hepatic maturation in 3D microenvironments.

Our molecular and functional analyses suggest that transitioning hepatic progenitors from 2D to 3D culture significantly enhances hepatocyte maturation across multiple dimensions. At the protein level, we observed markedly elevated expression of CPS1 and CYP3A4 in 3D-MH compared to their 2D counterparts, indicating increased activity of both the urea cycle and drug-metabolizing enzyme systems [29–30]. These findings were corroborated by gene expression analysis, which revealed significant upregulation of key mature hepatic markers including ALB, CPS1, AAT, and GLUT2 in 3D-MH. In addition to the increased expression of mature hepatic markers, 3D-MH cultures exhibited reduced CYP26A1 compared with 2D cultures. Although CYP26A1 is not a classical marker of hepatocyte maturation, it plays an important role in retinoic acid metabolism [31], and retinoic acid signaling is regulated during hepatocyte differentiation [32]. Therefore, the decreased expression of CYP26A1 is consistent with the more mature hepatic phenotype observed in the 3D cultures, further supporting the enhanced differentiation achieved using the 3D system. Conversely, FOXA2, a hepatic progenitor marker [13], showed markedly reduced gene expression and negligible protein levels in 3D-MH, likely reflectng the transition from a hepatic progenitor-like state toward a more differentiated hepatocyte. Functional assays further validated these molecular changes, demonstrating significantly enhanced albumin secretion, urea production, and glycogen storage in 3D cultures reflecting possible enhanced hepatic function. These improvements in synthetic and storage functions are consistent with previous reports [27, 28] showing that human iPSC-derived HLCs cultured as 3D-MH display enhanced albumin and urea secretion and increased increased cytochrome P450 (CYP1A2 and CYP3A4) activity compared with 2D cultures, collectively indicating a shift toward a more mature phenotype compared with traditional 2D culture systems.

Our comprehensive transcriptomic profiling analysis revealed distinct molecular signatures associated with each culture condition and provided critical insights into the biological pathways driving hepatocyte maturation. Pathway analysis demonstrated that genes upregulated in 3D-MH were predominantly involved in hepatic-specific metabolic and physiological processes, including pyruvate metabolism, drug metabolism, metabolism of xenobiotics by cytochrome P450, fatty acid degradation, retinol metabolism, glyoxylate and dicarboxylate metabolism, carbon metabolism, citrate cycle, glycine, serine and threonine metabolism. Consistent with this enhanced maturation, genes associated with the complement and coagulation, including C2, C3 and C7, were significantly upregulated indicating acquisition of specialized hepatic functions, which are the primary source of complement components and clotting factors [33–35]. Similarily, the upregulation of the metallothionein genes, *MT2A*,* MT2E*,* MT1M*,* MT1H*, and *MT1A*, suggests enhanced regulation of essential metal ions such as zinc, copper, and iron, which serve as vital cofactors for hepatic enzymes involved in detoxification and metabolic homeostasis [36–38]. Furthermore, the activation of bile secretion genes, such as *CYP7A1*,* CYP3A4*,* UGT2B4*, and *UGT2B17*, implies improved physiologic bile acid conversion from cholesterol and excretory function that are typically deficient in 2D cultures [39–42]. Furthermore, the robust induction of genes involved in xenobiotic metabolism, particularly *CYP3A4* and UGTs (*UGT2B15* and *UGT2B17*) underscores enhanced detoxification capacity in 3D-MH [43] aligning with our observed upregulation of CYP3A4 protein levels.

Validation of selected upregulated genes by RT-qPCR confirmed significant increases in the expression of transcription factors essential for hepatocyte identity and function, including ONECUT2, ONECUT3, PROX1, and FOXA3. These transcription factors play critical roles in hepatocyte differentiation, metabolic gene regulation, and maintenance of hepatocyte fate [44, 45]. In addition, we observed coordinated upregulation of genes encoding apolipoproteins (APOA2, APOC1, APOA5), additional cytochrome P450 enzymes (CYP3A5, CYP4A11), and metabolic enzymes involved in diverse pathways such as pyruvate carboxylase (PC), acetyl-CoA acetyltransferase 1 (ACAT1), acyl-CoA synthetase long-chain family member 4 (ACSL4), and various alcohol and aldehyde dehydrogenases (ADH6, ADH1A, ALDH4A1). The coordinated upregulation of these genes reflects a comprehensive metabolic maturation program activated in 3D culture, encompassing lipid metabolism, drug metabolism, and central carbon metabolism.

Conversely, genes downregulated in 3D-MH were enriched in pathways associated with focal adhesion, type 1 diabetes, hematopoietic cell lineage, cyokine-cyokine receptor interaction, JAK-STAT signaling pathway, and ECM-receptor interaction. This trend indicates that 2D-MH rely heavily on focal adhesion signaling for attachment and spreading on stiff substrates, whereas 3D-MH promotes cell to cell interactions and adhesion that are critical for hepatocyte maturation and maintenance of metabolic function [46]. These findings align with previous reports demonstrating that cells cultured in 3D conditions exhibit altered integrin signaling, reduced focal adhesion dependency, and distinct transcriptional states compared with 2D cultures [47]. Validation by RT-qPCR confirmed reduced expression of several genes associated with stemness and proliferation, including LIN28A, a marker of pluripotency and fetal-like states, as well as genes involved in cell adhesion (CADM1, CDH9) and ECM components (COL12A1, COL4A2). The concurrent downregulation of glucose transporters GLUT1 and GLUT3, which are typically expressed in proliferating and less differentiated cells, may further support the notion that 3D culture promotes exit from a progenitor-like state and progression toward terminal hepatocyte differentiation. The concordance between RNA-seq data, immunostaining, protein expression, and RT-qPCR validation underscores the robustness of our dataset and reinforces the conclusion that 3D culture conditions promote enhanced activation of metabolic, excretory, and immune-synthetic pathways, highlighting that 3D organization drives not only structural and morphological maturation but also restoration of complex functional characteristics associated with adult hepatocytes.

## Conclusion

This study suggest that transitioning from 2D to 3D culture at the hepatic progenitor stage enhances the maturation and functionality of iPSC-derived HLCs. Unlike previous studies that have primarily focused on phenotypic and functional characterization, our work uniquely combines analysis of mature hepatic marker expression, comprehensive functional assessment, and genome-wide transcriptomic profiling to provide mechanistic insights into the molecular programs and biological pathways that drive hepatocyte maturation in 3D microenvironments. Our findings reveal that 3D-MH not only exhibit enhanced expression of mature hepatocyte markers and metabolic functions but also activate a comprehensive suite of liver-specific transcriptional programs, associated with the enhancement of mitochondrial activity, oxidative metabolism, and hepatic detoxification capacity, which are hallmarks of functionally mature hepatocytes. By identifying molecular changes associated with 3D-induced hepatocyte maturation, our work may provide a basis for future optimization strategies and support ongoing efforts toward generating more mature, functional hepatocytes that more closely resemble the complexity and capabilities of the human liver for modeling human liver biology, screening hepatotoxic compounds, investigating drug metabolism pathways, and potentially developing next-generation cell-based therapies for liver disorders.

### Limitations of the study

While this study demonstrates the use of 3D culture in enhancing the functional maturation of iPSC-derived HLCs, several limitations should be acknowledged. The current study used a single iPSC line, and it is well established that differentiation efficiency and maturation capacity can vary considerably across different iPSC lines. Therefore, future studies should validate these results across multiple hiPSC and/or hESC lines to establish the reproducibility of the 3D culture system. Transcriptomic analyses revealed that 3D-MH have a more mature hepatic profile than 2D-MH, including downregulation of progenitor and fetal markers (*PROM1*,* LIN28A*, and *GLUT1/3*) [48, 49] and a closer resemblance to adult human liver tissue, but low level expression of pancreatic-associated genes such as PAX4 and GCG, as well as CD4, was also detected. These transcripts are expressed at low relative levels and likely reflect residual developmental plasticity, non-specific transcription, or shared developmental origin of liver and pancreas rather than true lineage contamination. However, current iPSC-derived HLC differentiation protocols are known to retain residual fetal-like or mixed-lineage transcriptional signatures [11], suggesting that the adult hepatocyte transcriptome has not been fully recapitulated. Furthermore, although comparison to publicly available GTEx adult liver transcriptomes suggested that 3D-MH were transcriptionally more similar to adult liver tissue than 2D-MH, a side-by-side comparison with freshly isolated primary human hepatocytes under identical experimental conditions was not performed. Similarly, although maturation is improved in the current study, additional functional characterization to the level of adult primary hepatocytes is required to fully define the extent of functional equivalence. Future studies with single-cell RNA sequencing will continue to resolve cellular heterogeneity and lineage purity, and expanded functional benchmarking against primary human hepatocytes will afford a more comprehensive assessment of the utility of 3D-derived HLCs for disease modelling, drug screening, and regenerative medicine applications.

## Supplementary Information

Below is the link to the electronic supplementary material.

Supplementary Material 1

Supplementary Material 2

Supplementary Material 3

Supplementary Material 4

Supplementary Material 5

## Data Availability

The data that support the findings of this study are available from the corresponding author upon reasonable request.
