# Supplementary material for: Three-dimensional organoid culture enhances functional maturation of human pluripotent stem cell–derived hepatocytes
Source: Mol Biol Rep. 2026 Jul 29;53(1):1294. doi: 10.1007/s11033-026-12480-9 (PMC13421251; doi:10.1007/s11033-026-12480-9)
Supplement: Supplementary file 1 — Supplementary Material 1 [file 11033_2026_12480_MOESM1_ESM.pdf]

Supplementary Figures

Supplementary Figure 1

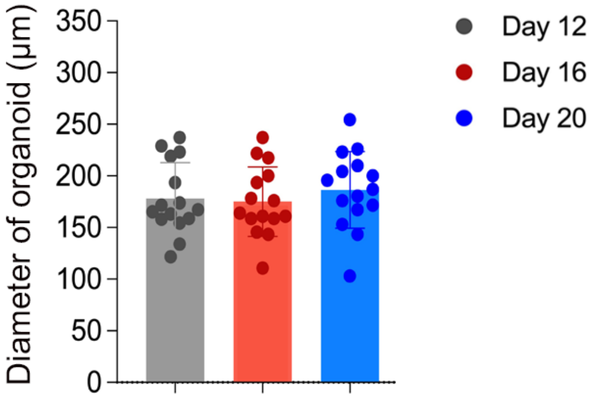

Supplementary Figure 1. Quantitative analysis of organoid diameter (n = 15 organoids per group) at days 12, 16, and 20 of 3D-MH culture. No significant differences in organoid diameter were observed across the analyzed time points, indicating stable organoid size throughout the culture period.

Supplementary Figure 2

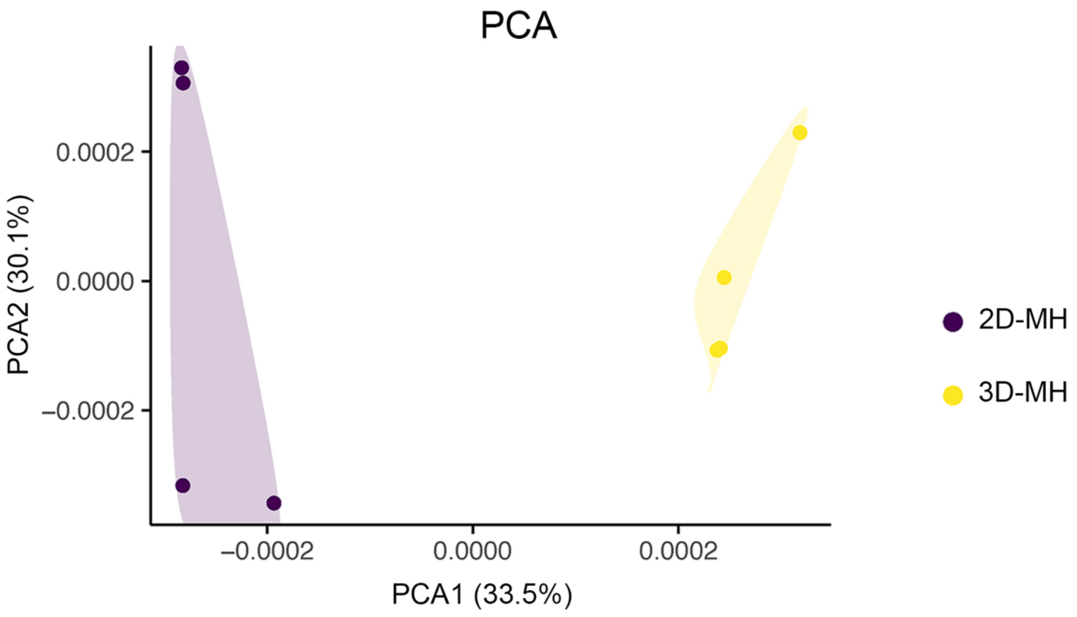

Supplementary Figure 2. Global transcriptomic variation between 2D-MH and 3D-MH assessed by Principal Component Analysis (PCA). Each data point represents an individual sample colored by conditions (2D-MH and 3D-MH). PC1 and PC2 explain 33.5% and 30.1% of the total variance, respectively. PERMANOVA indicated significant separation between conditions (adonis $R^2 = 0.33$, $P = 0.031$), with homogeneous group dispersion (betadisper $P = 0.488$). Plot generated using ImageGP.
