# Supplementary material for: Three-dimensional organoid culture enhances functional maturation of human pluripotent stem cell–derived hepatocytes
Source: Mol Biol Rep. 2026 Jul 29;53(1):1294. doi: 10.1007/s11033-026-12480-9 (PMC13421251; doi:10.1007/s11033-026-12480-9)
Supplement: Supplementary file 2 — Supplementary Material 2 [file 11033_2026_12480_MOESM2_ESM.docx]

**Supplementary Table 1.** *In vitro* differentiation of iPSCs into hepatocytes

| **Stage of differentiation** | **Basal media** | **Cytokine concentration** |
| --- | --- | --- |
| Stage 1: Definitive Endoderm (Day 1) | MCDB131  1% Pen/Strep  1% L-Glutamine  10 mM Glucose  0.5% BSA  1.5 g/L NaHCO3 | 100 ng/mL Activin A  0.25 mM Vitamin C  2 µM CHIR99021  10 µM Rock Inhibitor |
| Stage 1: Definitive Endoderm (Day 2 – Day 4) | MCDB131  1% Pen/Strep  1% L-Glutamine  10 mM Glucose  0.5% BSA  1.5 g/L NaHCO3 | 100 ng/mL Activin A  0.25 mM Vitamin C |
| Stage 2: Posterior Foregut  (Day 5) | MCDB131  1% Pen/Strep  1% L-Glutamine  10 mM Glucose  0.5% BSA  1.5 g/L NaHCO3 | 10 ng/mL FGF2  30 ng/mL BMP4  2 µM Retinoic Acid  10 μM SB431542  10 µM Rock Inhibitor |
| Stage 2: Posterior Foregut  (Day 6) | MCDB131  1% Pen/Strep  1% L-Glutamine  10 mM Glucose  0.5% BSA  1.5 g/L NaHCO3 | 10 ng/mL FGF2  30 ng/mL BMP4  10 μM SB431542 |
| Stage 3: Hepatic Progenitor (Day 7 – Day 8) | DMEM – F12  1% Pen/Strep  1% L-Glutamine  20% Knockout Serum Replacement  1% Non-Essential Amino Acids | 10 ng/mL Activin A  30 ng/mL BMP4  1µM Forskolin |
| Stage 3: Hepatic Progenitor (Day 9) | DMEM – F12  1% Pen/Strep  1% L-Glutamine  20% Knockout Serum Replacement  1% Non-Essential Amino Acids | 10 ng/mL Activin A  30 ng/mL BMP4  1 µM Forskolin  1 µM CHIR99021 |
| Stage 4: Hepatic Mature  (Day 10 – Day 11) | DMEM – F12  1% Pen/Strep  1% L-Glutamine  0.5% Non-Essential Amino Acids | 10 ng/mL BMP4  10 ng/mL Oncostatin  10 mg/L ITS  200 µg/mL Vitamin C  2 µM Gamma-Secretase Inhibitor  10 µM Dexamethasone  1 µM SB431542 |
| Stage 4: Hepatic Mature  (Day 12 – Day 15) | DMEM – F12  1% Pen/Strep  1% L-Glutamine  0.5% Non-Essential Amino Acids | 10 ng/mL BMP4  10 ng/mL Oncostatin  10 mg/L ITS  200 µg/mL Vitamin C  2 µM Gamma-Secretase Inhibitor  10 µM Dexamethasone |
| Stage 4: Hepatic Mature  (Day 16 – Day 21) | DMEM – F12  1% Pen/Strep  1% L-Glutamine  0.5% Non-Essential Amino Acids | 10 mg/L ITS  200 µg/mL Vitamin C  2 µM Gamma-Secretase Inhibitor  10 µM Dexamethasone  1 µM Forskolin |
