# Supplementary material for: Three-dimensional organoid culture enhances functional maturation of human pluripotent stem cell–derived hepatocytes
Source: Mol Biol Rep. 2026 Jul 29;53(1):1294. doi: 10.1007/s11033-026-12480-9 (PMC13421251; doi:10.1007/s11033-026-12480-9)
Supplement: Supplementary file 3 — Supplementary Material 3 [file 11033_2026_12480_MOESM3_ESM.docx]

**Supplementary Table 2.** List of antibodies used for immunofluorescence and western blotting

| **Antibody** | **Company** | **Catalog #** | **Dilution** |
| --- | --- | --- | --- |
| β-actin | Santa Cruz | sc-47778 | WB (1:10,000) |
| GAPDH | Cell Signaling | 5174s | WB (1:5000) |
| Alpha tubulin | Abcam | ab24610 | WB (1:5000) |
| CPS1 | Abcam | ab45956 | WB (1:1000)  IF (1:500) |
| CYP3A4 | Abcam | ab135813 | IF (1:500) |
| HNF4a | Cell Signaling | 3113S | IF (1:500) |
| Albumin | Sigma | A6684 | WB (1:5000)  IF (1:500) |
| FOXA2 | Cell Signaling | 3143 | WB (1:4000)  IF (1:1000) |
| AFP | Abcam | ab3980 | IF (1:2000) |
| 594 donkey anti-rabbit | Invitrogen | A21207 | IF (1:500) |
| 488 donkey anti-rabbit | Invitrogen | A21206 | IF (1:500) |
| 594 Donkey anti- mouse | Invitrogen | A21203 | IF (1:500) |
| 488 donkey anti-mouse | Invitrogen | A21202 | IF (1:500) |
| Peroxidase AffiniPure Donkey anti-Rabbit IgG (H+L) | Jackson ImmunoResearch Laboratories | 711-035-152 | WB (1:10,000) |
| Peroxidase AffiniPure Donkey anti-Mouse IgG (H+L) | Jackson ImmunoResearch Laboratories | 715-035-150 | WB (1:10,000) |
