# Supplementary material for: Three-dimensional organoid culture enhances functional maturation of human pluripotent stem cell–derived hepatocytes
Source: Mol Biol Rep. 2026 Jul 29;53(1):1294. doi: 10.1007/s11033-026-12480-9 (PMC13421251; doi:10.1007/s11033-026-12480-9)
Supplement: Supplementary file 4 — Supplementary Material 4 [file 11033_2026_12480_MOESM4_ESM.docx]

**Supplementary Table 3.** List of primers used for qPCR analysis

| **Gene Name** | **Forward primer sequence** | **Reverse primer sequence** |
| --- | --- | --- |
| *ALB* | GTGAAACACAAGCCCAAGGCAACA | TCAGCCTTGCAGCACTTCTCTACA |
| *CPS1* | TTTCAAAGTGGTGAGGACACTG | TCTTAGTTCCATCTTCCAGGAC |
| *CYP3A5* | CACAGAACACAGTTGAAGAAGG | TACGGGTCCCATATAGATAGAGG |
| *CYP26A1* | GAAACCTTGCAGATGGTACTG | CGAACAGATGCGTCTTGTAGATG |
| *CYP4A11* | ATTCAGAAATGGGTGGAGACAT | ATGGGATTTCGGGTCTGATC |
| *PROX1* | AAAGTCAAATGTACTCCGCAAGC | CTGGGAAATTATGGTTGCTCCT |
| *GLUT1* | CTGCTCATCAACCGCAAC | CTTCTTCTCCCGCATCATCT |
| *GLUT2* | ATGAACTGCCCACAATCTCATA | GGACCAGAGCATGGTGATTAG |
| *GLUT3* | CAGCGAGACCCAGAGATG | TTGGAAAGAGCCGATTGTAG |
| *AAT* | AGGTGCCTATGATGAAGCGT | TGGCAGACCTTCTGTCTTCATT |
| *APOA2* | GTTCGGAGACAGGCAAAGGA | TCAAAGTAAGACTTGGCCTCGG |
| *APOA5* | AAGCAGAATGTCTGCTCTCTGTG | AAACGCTGAAAGAAGAGCCAGAG |
| *ONECUT2* | ACTGTTCTCTGAGCATGCTAAG | AGCTTGGTTGACACCATACTG |
| *ONECUT3* | CGTCAGCAACTTCTTCATGAACG | CTCAGGCCTTGGAGAAAGTG |
| *FOXA2* | GGGAGCGGTGAAGATGGA | TCATGTTGCTCACGGAGGAGTA |
| *FOXA3* | TAACATCTGGGTGGGTCT | CAGTGGATTAGCCAATAACA |
| *LPA* | GCCAGTCCCAAAATGGA | TGCTCAGGTGCTGCTGATTTC |
| *ACAT1* | CGATACTCAGCCCTCTGCG | TTATTTCCTGCACCAGCCTCC |
| *ACSL4* | GGAGGGAATGTCCGCATGAT | CTGTCCCAGCACCACATGAT |
| *ADH1A* | TCTGGGAAAAGTATCCGTACCATT | TGAAGACTGCCACAAGGGAA |
| *ALDH4A1* | CCCGCTTCTAACCCGAGATG | CCCTGCGTGAAGGCTAAGAC |
| *FAHD1* | ATACACGGGCTGGTCAGTATG | CCGTTGCTTGCTCTTGTCTTG |
| *MT1E* | GCTTGTTCGTCTCACTGGTG | TTGCAGGAGGTGCATTTG |
| *MT1G* | CCACTGCCTCTTCCCTTCTC | GAGACACCAGCGGCACAG |
| *MT2A* | CCTCCTCCAAGTCCCAGC | ATCCATGGCGAGCTGAAGAG |
| *PC* | CCAGATGCACCGGCAGAAAG | GCTCAGAGAGGAACCCGTAG |
| *HAMP* | TTTTCCCACAACAGACGGGA | CTCCTTCGCCTCTGGAACAT |
| *NPY* | CGCTGCGACACTACATCAACC | AGGGTCTTCAAGCCGAGTTCTG |
| *CADM1* | ACATGGCGAGTGTAGTGCTG | CCATCACCTGTGGGGATCAG |
| *CDH9* | GACTGTGCCTCTGTGGGAAA | TGGGTCTGCGTCGTTTTCTT |
| *CDH3* | TGACCACAAGCCCAAGTTTAC | TAAGCAACCACCCCATTGTAG |
| *COL12A1* | CACAAGCTCCCAAAAGTGGC | TCCCCTGTGGAAGGCTGATA |
| *COL4A2* | CCTGAAGGCACAGCTAACCA | TGCTGTTGTCTCGTCTGTCC |
| *CPE* | TGAGGCTGTTGGACGAGAAC | GTGAATGCGGGTACTGTGGA |
| *LAMB1* | CGGAAAGGAAGACGGGAAG | AACTGGAGAAGCCCCATGC |
| *ADH6* | GCCCCACCAAAGGCAAAG | CTCAACGATTCCAGCCCCTT |
| *APOC1* | AGCCGCATCAAACAGAGTGA | TCCTGGGATGTCACCCTTCA |
| *ARG1* | GTCTGTGGGAAAAGCAAGCG | CACCAGGCTGATTCTTCCGT |
| *C1S* | GGTGGGGAGTATCACTGTGC | CTGGAACACATTTCGGCAGC |
| *GC* | AGTGGCACGTTTGAACAGGT | CATAGCAGTCAGGGTCAGCC |
| *GCGR* | AGGTGATGGACTTCCTGTTTGAG | TACTTGTCGAAGGTTCTGTTGC |
| *PLG* | GCTGGGAGCAGGAAGTATAG | AGCCATTATCACACATTGTTGC |
| *UGT2B15* | TCAGTGTGGACATCAGGACCAT | CGATCCAGGGGCTTCATTGGT |
| *UGT2B4* | AGACGTTGTGATACAAGAGATTCCT | TCCCTAATGTTTTCCTCCTTGACA |
| *UGT2B17* | TCCAAAAGTTACCCCACACAA | TGGAGTTGTTAATGTTTTGTGTTCA |
| *TLN2* | ATTGTTGCCAAGCACACGTC | GGCTGACTGGACGAAGT |
| *NOG* | TAGCTTTCTGGTTCCTGTAATG | ACAGTAGAAGCCGGTAACT |
| *IRX1* | CAAGAATCCCTACCCCACCA | TCCCCATGTCACCTTGTTCT |
| *LIN28A* | GGTGCGGGCATCTGTAAGTG | GGAACCCTTCCATGTGCAGC |
| *PRSS12* | GCCACAGAAAACAGCATCCA | CTCCCTGTAAACCGACCCTT |
